# Impact of Extraction Parameters on the Gallic Acid Content and Antioxidant Properties of Palo Prieto (*Lysiloma divaricata*) Fractions and Their Identification via UPLC-MS/MS

**DOI:** 10.3390/antiox14091074

**Published:** 2025-09-01

**Authors:** Daniela Gómez-Espinoza, J. A. Gonzalez-Calderon, Enrique Delgado-Alvarado, Agustín L. Herrera-May, Leandro García-González, César Leobardo Aguirre-Mancilla, Ricardo Rivera-Vázquez, Ma. Cristina Irma Pérez-Pérez

**Affiliations:** 1 Departamento de Ingeniería Bioquímica y Ambiental, TecNM en Celaya, Celaya 38010, Guanajuato, Mexico; d2303031@itcelaya.edu.mx; 2 Cátedras SECIHTI–Instituto de Física, Universidad Autónoma de San Luis Potosí, San Luis Potosí 78290, San Luis Potosí, Mexico; amir.gonzalez.itc@gmail.com; 3 Micro and Nanotechnology Research Center, Universidad Veracruzana, Boca del Río 94294, Veracruz, Mexico; endelgado@uv.mx (E.D.-A.); leherrera@uv.mx (A.L.H.-M.); leagarcia@uv.mx (L.G.-G.); 4 Facultad de Ingeniería de la Construcción y el Hábitat, Universidad Veracruzana, Boca del Río 94294, Vercaruz, Mexico; 5 Tecnológico Nacional de Mexico/IT de Roque, Celaya 38110, Guanajuato, Mexico; cesar.am@roque.tecnm.mx; 6 Instituto Nacional de Investigaciones Forestales, Agrícolas y Pecuarias, Campo Experimental Bajío, Celaya 38110, Guanajuato, Mexico; rivera.ricardo@inifap.gob.mx

**Keywords:** antioxidant compounds, extraction methods, Lysiloma divaricata, gallic acid, phenol compounds

## Abstract

The palo prieto (*Lysiloma divaricata*) is a tree with grayish bark and pinnate leaves that is native to Mexico. This tree can reach heights close to 15 m and is a source of phytochemical compounds, including polyphenols. The optimized extraction method is important for preserving phytochemical compounds, particularly gallic acid. In general, solid-liquid extraction methods are the most commonly used methods for obtaining phytochemical compounds from *Lysiloma divaricata*. Herein, we report the results of a complex experimental design in which different parts of the plant (leaf, stem, and fruit) were used to investigate their antioxidant activities and gallic acid contents. In this design, we included variations in the type of solvent, time, and temperature. This method yields an extract rich in phytochemical components that may exhibit significant antioxidant activity, making it suitable for isolating natural antioxidant compounds. For these compounds, bromatological analysis, quantification of phenolic content, and identification and quantification of phytochemical compounds via UPLC-MS/MS identified 27 compounds, with gallic epicatechin, catechin, kaemferol-3-glucoside, procyanidin B1, and gallic acid as the major compounds. For the quantification of gallic acid by HPLC, the highest concentration of gallic acid was detected in the water-leaf-40 °C-90 min fraction. In addition, antioxidant activity via 1,1-diphenyl-1,2-picrylhydrazyl (DPPH), 2,2-azinobis (3-ethylbenzothiazoline-6-sulfonic acid) (ABTS), and ferric reducing antioxidant power (FRAP) was studied, and color measurements were performed. Additionally, the antioxidant activity of the fruit samples was evaluated via the DPPH method with an ethanol/water ratio of 30:70 % v/v at 60 °C for 60 min, which resulted in the highest percentage of inhibition. There was no significant difference in the antioxidant activity when ABTS was used between the samples. For the antioxidant activity determined via FRAP, the leaf sample exhibited the most significant activity when ethanol was used as the solvent at 50 °C for 90 min, with a value of 195,861 ± 44.20 µM eq Trolox/g DM. The phenol compounds of *Lysiloma divaricata* are promising sources of natural antimicrobials and antioxidants for potential applications in food packaging.

## 1. Introduction

The *Lysiloma divaricata* tree is known as the palo prieto, tepeguaje [1] or quebracho liso [2]. This tree reaches a height between 3 and 15 m and has grayish bark with scales and no thorns, with foliar inflorescences that are globose in shape [3]. This plant is used in traditional Mexican medicine in certain regions where it grows. For this reason, more investigations into the phytochemical compounds of this plant are needed. *Lysiloma divaricata* is a rich source of phytochemical compounds, including polyphenols [4]. Phytochemical compounds are secondary metabolites synthesized by plants. These compounds protect plants from both biotic and abiotic stresses. In addition, they provide color and flavor to fruits and vegetables [5]. Phytochemicals can include polyphenols, alkaloids, terpenes, saponins, coumarins and flavonoids [6]. Polyphenolic compounds consist of aromatic rings with hydroxyl groups, organic acids, and sugars. These phenolic fractions have high antioxidant activity that prevents the formation of free radicals [7].

An antioxidant is a molecule capable of inhibiting the oxidation of other molecules [8]. It is a substance that delays, prevents, or eliminates oxidative damage to a target molecule. Antioxidants are compounds that can inhibit reactive oxygen species and free radicals. Thus, antioxidants can help in the treatment of carcinogenesis, cardiovascular diseases and aging [9]. The chemical structures and compositions of phenolic compounds in plants are very complex, resulting in high variation in their antioxidant activities. Several factors can affect the antioxidant activity of phenolic compounds, such as their source, chemical structure, molecular weight, purity and processing technology [10]. Phenolic acids are usually subclassified into hydroxybenzoic acid derivatives and hydroxycinnamic acid derivatives [11]. Hydroxybenzoic acid derivatives include gallic acid (3,4,5-trihydroxybenzoic acid), which is currently considered one of the main phenolic acids due to its antioxidant activity [12]. It can be found in the structure of different phytochemical compounds, mainly as part of hydrolysable tannins. It has anticancer, anti-HIV, antiulcer, anti-inflammatory, antimicrobial, and antifungal properties [13], as well as antidiabetic properties [14].

The method of extracting this compound is important to ensure its preservation. Liquid-liquid and solid-liquid extraction methods are commonly used for phenolic acids and flavonoids [15]. The simplest method of solid-liquid extraction is single-contact batch operation. This method can be implemented in two steps: contacting the solvent and the solid material to transfer the solute into the solvent and then separating the solution from the remaining solid [16]. This process can be performed via a rotary evaporator. The extraction efficiency is determined by several critical elements, including the extraction technique, the matrix properties of the plant components, the extraction solvent, temperature, pressure, and time [17]. The most commonly employed extraction solvents are alcohols (methanol, ethanol), acetone, diethyl ether and ethyl acetate. However, highly polar phenolic acids (benzoic and cinnamic acids) cannot be completely extracted with pure organic solvents [18]. Solvent mixing can improve extraction performance, and alcohol-water mixtures are recommended. The properties of extraction solvents, such as polarity, can be adjusted according to the proportion of solvents [19]. After the extraction process, in vitro test methods must be applied to evaluate the antioxidant capacity of the extracted phytochemical compounds. Various methods must be used to consider the following factors: the diversity and complexity of the antioxidant reaction mechanism, the distribution effect of antioxidants in a heterogeneous system and the influence of other components in the test system [20]. Therefore, several methods have been employed to measure the antioxidant capacity of phytochemical compounds. Some of these methods involve 2,2-diphenyl-1-picrylhydrazyl (DPPH), 2,2′-azino-bis-(3-ethylbenzthiazoline-6-sulfonic acid) (ABTS), and ferric reducing antioxidant power (FRAP). These reaction principles are essentially based on the scanning capacity of artificially produced free radicals to evaluate the antioxidant capacity of the substance under test [21].

In this work, we present the results of a complex experimental design to investigate the gallic acid content and antioxidant activity of different parts (leaf, fruit, and stem) of a *Lysiloma divaricata*. In this design, we assess the influence of variations in time, temperature, and solvent type. Thus, the optimal conditions for extracting phytochemicals have been determined. Furthermore, the plant part with the greatest amount of these compounds was identified. Additionally, we report high-performance liquid chromatography (HPLC) analysis to identify the gallic acid content in leaf, fruit, and stem samples with different solvents, including those with lower and higher gallic acid equivalent concentrations.

## 2. Materials and Methods

2,2-Diphenyl-1-picrylhydrazyl (DPPH), Lot 102703519, CAS 1898-66-4, Sigma Aldrich, Buchs, Switzerland, ABTS Lot 12774733, CAS 10102946001, Roche, Mannhein, Germany, Folin–Ciocalteu Lot 390753 brand Hycel, CAS 76969-54-5, USA, Trolox Lot 102703519, Sigma Aldrich, CAS 53188-07-1, Buchs, Switzerland, TPT2 (2,4,6-Tris (2-pyridyl)-s-triazine), brand Sigma Aldrich, and Lot 102656107, CAS 3682-35-7, Buchs, Switzerland, Methyl alcohol, 99.8%, CAS 67-56-1, Karal brand, Lot. 36852, Leon, Gto, Mexico, absolute ethyl alcohol, 99.5%, CAS 64-17-5, Karal brand, Lot 36266, Leon, Gto, Mexico, Ecopure water, reagent grade, Mexico. The fruits, leaves, and stems of *Lysiloma divaricata* were obtained from the National Institute of Forestry, Agricultural and Livestock Research (INIFAP) located in Celaya, Guanajuato, Mexico (at the geographic coordinates 20°32′05″ N and 100°48′ 49” W, at an altitude of 1750 m above sea level), during the period from November 2023 to March 2024. The collected leaves, fruits and stems were dried at 40 ± 5 °C (7 kg of leaves, 2 kg of fruit and 10 kg of stem were used). The stems were dried in an oven for 7 days, while the leaves and fruits were dried for 5 days. After drying, the samples were ground and sifted through a sieve with mesh No. 30. Finally, the processed samples were packed in bags, sealed tightly and stored.

### 2.1. Bromatological Analysis

Bromatological analysis of the plants was performed to determine their chemical composition and evaluate their functional value. In this case, the bromatological analysis was completed in triplicate according to the AOAC [22]. The parameters studied were humidity (AOAC 14.003), ash content (AOAC 14.006), lipid content (AOAC 7.062), protein content (AOAC 2.057), and crude fiber content (AOAC 962.09). Carbohydrates were determined on the basis of differences in accordance with the recommendations of the FAO [23] from the results obtained in the determinations of lipids (L), ash (A), protein (P), moisture (M), and fiber (F). Thus, the percentage of carbohydrates was calculated as follows:

Carbohydrates (%) = 100 − (F + A + P + M + L)(1)

### 2.2. Fourier Transform Infrared Spectroscopy (FTIR)

The sample was placed in a closed cell isolated from the environment. Infrared spectra were recorded using a Perkin-Elmer model Spectrum 100 spectrometer. The spectral range was scanned from 400 to 4000 cm^−1^. Each measurement was made with 25 scans.

### 2.3. Extraction Procedure

The methodology of Pérez et al. [24] was used with some modifications. The sample was extracted following a 3^3^ design, taking three extraction times 30, 60, and 90 min, using three solvents, ethanol/water 30:70 % v/v, methanol/water 30:70 % v/v and 100% water, were used in a rotary evaporator at three different temperatures, 40°, 50°, and 60 °C, to determine the optimum temperature, and the best solvent and tree fraction to work was obtained.

The proportion of extraction solution and the sample of *Lysiloma divaricata* powder was 2.6 g/100 mL, and the total phenols of the extract were measured to determine the optimal extraction time of the compounds. After extraction, the samples were filtered through Whatman filter paper No. 4.

### 2.4. Determination of Total Phenolic Content

The phenolic content was determined according to the previous method reported in the literature by Soria-Melgarejo et al. [25], with some modifications. A total of 25 μL of the sample, 25 μL of Folin–Ciocalteu reagent, and 25 μL of sodium carbonate were added to a microplate. The mixture was incubated for 30 min at 40 °C, 200 μL of distilled water was added, and the absorbance was read at 750 nm. The calibration curve was generated with a gallic acid (GA) solution. The results are expressed in mg of gallic acid equivalents per gram of dry weight of the sample in mg of gallic acid equivalents (mg GAE/g dry matter (DM)). The following equation was used:

(mg GAE)/(g dry matter (DM)) = ((mg/mL) × (v extract (mL))/(g sample)(2)

### 2.5. Ultra-High-Resolution Chromatography Coupled with Tandem Mass Spectrometry (UPLC-MS/MS)

The analysis was carried out via an UPLC system (Acquity Class-H, Waters Corp., Milford, MA, USA) coupled to a triple quadrupole mass spectrometer (Xevo TQ-S, Waters Corp., Milford, MA, USA). The system included a sample handler operated at 6 °C and a quaternary pump. A reversed-phase Acquity^®^ BEH C18 column with a particle size of 1.7 µm and dimensions of 50 mm × 2.1 mm ID (Waters Corp., Milford, MA, USA) operating at 35 °C and a flow rate of 0.25 mL/ min was used for the separation of phenolic compounds. A binary solvent system was utilized, consisting of 7.5 mM formic acid in Milli-Q water (referred to as solvent A) and acetonitrile (referred to as solvent B). The system was set to a step gradient of 3, 9, 16, 50, and 3% solvent B at 0, 1.23, 3.82, 11.40, and 13.24 min, respectively. The mixture was stabilized for 2.76 min with 3% solvent B. Electrospray ionization (ESI) was conducted in negative mode with the following settings: nitrogen gas was used as a nebulizer, the ESI source was set at 2.5 kV, the collision gas flow was 0.13 mL per min, the MS mode collision energy was 5.0, and the MS/ MS mode collision energy was 20.0. The desolvation and source temperatures were set at 400 °C and 150 °C, respectively. For the carbohydrate profile, an Acquity BEH Amide column (1.7 μm particle size, 100 mm × 2.1 mm ID; Waters Corp, Milford, MA, USA, EE.UU.) was used at 35 °C. A binary solvent system was utilized, consisting of an acetonitrile/water 0.1% NH_4_OH solution (80:20) (referred to as solvent A) and an acetonitrile/water 0.1 % NH_4_OH solution (referred to as solvent B). The gradient began with 0% B, increased to 60% B at 5 min, then was reset to 0% B at 10 min, and the column was stabilized for another minute. The flow rate was set at 170 μL/min, and the injection volume was 0.1 μL. Electrospray ionization (ESI) was conducted in negative mode with the following settings: capillary voltage of 2500 V, desolvation temperature of 300 °C, source temperature of 120 °C, desolvation gas flow of 500 L/h, cone gas flow of 150 L/h, collision gas flow of 0.13 mL/min, MS collision energy mode of 2.0, and MS/MS of 20. The retention time was utilized, and the *m*/*z* values along with the MS/ MS transitions of phenolic compounds were tracked via multiple reaction monitoring (MRM) on both samples and standards [26].

### 2.6. Chromatographic Parameters

The separation of gallic acid was performed by liquid chromatography (HPLC) with an Agilent 1200 series with a diode array detector (UV VIS). Column: Discovery HS C-18 (250 mm × 4.6 mm, 5 µm). Mobile phase: 26:74 (acetonitrile/water pH 2.66 with phosphoric acid). The flow rate was 1.0 mL/min, the injection volume was 10 µL, the column temperature was 28 °C, the wavelength was 272 nm, the run time was 9 min, and the retention time of gallic acid was 2.8 min.

### 2.7. Assays Used for Determination of Antioxidant Activity

#### 2.7.1. Determination of Antioxidant Activity with DPPH

The DPPH method was evaluated according to the procedure proposed by Benzie & Strain [27]. The reading was performed in a 96-well plate, where 20 µL of extract and 280 µL of radical were added for the measurement of the sample. In addition, 20 µL of absolute methanol and 280 µL of radical were used as targets. After 30 min, the mixture was allowed to rest in the absence of light, and the reading was taken at 515 nm. The results are expressed as % inhibition. The following equation was used:

% inhibition = ((Abs. sample − Abs. blank)/(Abs. sample)) × 100(3)

#### 2.7.2. Determination of Antioxidant Activity via FRAP

The FRAP method was evaluated according to the procedure proposed by García-Hernández et al. [28]. In a 96-well microplate, 20 µL of extract and 280 µL of radical were added, and the mixture was left to stand for 30 min in the absence of light; the absorbance was read at 593 nm. The calibration curve was generated with an 800 µM Trolox solution. The results are expressed as mg of Trolox equivalents per gram of dry weight of the sample (mg Trolox/g DM). Equation (4) was used.

(µM Trolox equivalents/g) = ((mg/mL) (v extract(mL))/(g sample))(4)

#### 2.7.3. Determination of Antioxidant Activity with ABTS

The ABTS method was evaluated according to the procedure proposed by Brand-Williams et al. [29]. The reading was performed in a 96-well plate, in which 20 µL of Trolox + 230 µL of the working solution were added for the measurement of the sample. The mixture was allowed to rest for 6 min, after which the absorbance was read at 734 nm. The samples were read at 4, 10, 30, 60, and 90 min. A calibration curve was generated with 800 µM Trolox. The results are expressed as % inhibition. To obtain these results, Equation (3) was used.

### 2.8. Determination of Color

Color determination was carried out using the Chroma Meter CR-400 (Konica Minolta, Morristown, NJ, USA), and the color coordinates were evaluated according to the scale proposed by the CIE L a b chromaticity and hue degrees.

### 2.9. Statistical Analysis

The results are expressed as the mean ± standard deviation of the experiments with three repetitions. Statistical evaluation was performed via analysis of variance (ANOVA), comparing the means via the Tukey method with a significance level of *p* ˂ 0.05, using the Statgraphics centurion XVI program.

## 3. Results

The results obtained from the bromatological analysis of the three samples (leaf, fruit and stem) of *Lysiloma divaricata* are presented in Table 1. This table shows the moisture, lipid, ash, protein, fiber and carbohydrate contents of each of the three samples. There was no significant difference in the percentage of moisture. On the other hand, the leaves presented the highest percentage of ash, indicating a relatively high inorganic content, such as minerals. The results of the bromatological analysis of this work, compared with those of other species of *Lysiloma*, show, for example, that the leaves of *Lysiloma acapulcensis* can have a protein content of 17.70% dry matter (DM) [30], whereas *Lysiloma latisiliquum* has achieved 47.10% moisture, 21.30% crude protein, and 7.90% ash in its leaves and 50.80% moisture, 8.80% protein, and 7.20% ash in its stems [31]. In comparison, the protein values obtained for *Lysiloma divaricata* were lower. The ash content was also lower in the fruit and stem fractions. However, the leaf fraction of *Lysiloma divaricata* has a greater percentage of ash than that of *Lysiloma latisiliquum*.

### 3.1. Fourier Transform Infrared Spectroscopy (FTIR)

**Figure 1:**
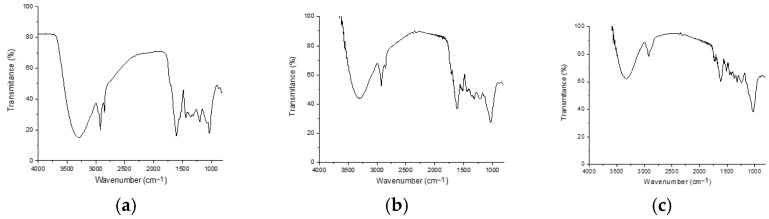
FTIR spectra of samples of (**a**) leaves, (**b**) fruits, and (**c**) stems of *Lysiloma divaricata*.

Bands 3295, 3307, and 3334 present in each of the FTIR spectra can be associated with water, whereas bands 2917, 2918, 2918, 2918, 2851, and 2851 are associated with symmetric stretching of CH groups [32]. The bands at 1433 cm^−1^ are related to carbonyl groups associated with secondary amides [33]. The 1610–1630 cm^−1^ peaks are related to hydroxyl (Si-OH) bonds. Bands at 1220–1260 cm^−1^ are also visualized for fruits and stems, which could correspond to fucoidans [34]. The peaks between 1100 and 1000 cm^−1^ (1025, 1038, and 1019 cm^−1^) originate from the CO stretching characteristic of cell wall carbohydrates (alginates, fucoidans and cellulose) and reserve carbohydrates (laminarin and mannitol) [35]. For stem 1728, a shoulder peak at 1728 cm^−1^ and an ester group vibration peak [36] are shown in Figure 1.

### 3.2. Phenolic Content

**Figure 2:**
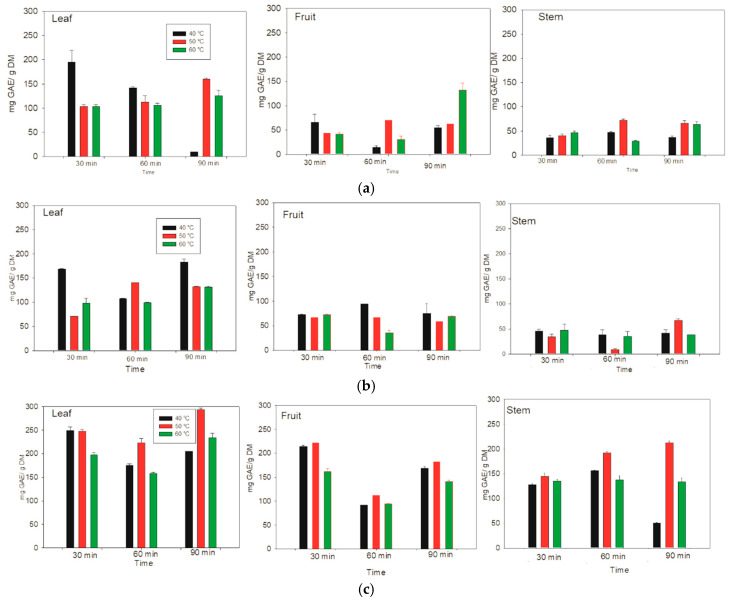
Phenolic contents of the leaves, fruits, and stems of *Lysiloma divaricata* at three temperatures (40 °C, 50 °C and 60 °C), three times (30 min, 60 min, 90 min), and three solvents: (**a**) water, (**b**) ethanol/water (30:70 % v/v), and (**c**) methanol/water (30:70 % v/v).

The phenolic contents of the aqueous, methanolic and ethanolic extracts were quantified in the leaves, fruits, and stems of *Lysiloma divaricata* at different temperatures (Figure 2). For the aqueous extract of the leaf, the optimal extraction of 195.31 ± 23.51 mg GAE/g DM was obtained at 40 °C for 30 min. In addition, the optimal extraction for fruit was 132.48 ± 14.41 mg GAE/g DM at 60 °C for 90 min. Finally, the optimal extraction for stems was 29.26 ± 1.76 mg GAE/g DM at 60 °C, and better extractions were obtained at 60 °C. However, for the leaves of *Lysiloma divaricata*, the temperature and time were relatively low, making it the optimal extraction model for aqueous systems.

For the leaves of *Lysiloma divaricata*, the optimal extraction of phenolic compounds content in the ethanolic extract was 168.70 ± 1.02 mg GAE/g DM at 40 °C for 30 min. For the fruits and stems, the optimal extractions were 72.57 ± 0.81 mg GAE/g DM and 66.46 ± 3.99 mg GAE/g DM at 40 °C and 50 °C for 60 min and 90 min, respectively. Thus, the optimal extraction temperature was 40 °C, varying the times. However, for the leaves, the optimal extraction results were obtained at relatively low temperatures and times, making it the optimal extraction model for the ethanolic system. The optimal extraction of phenolic compounds content in the methanolic extract of *Lysiloma divaricata* leaves was 248.94 ± 7.63 mg GAE/g DM at 40 °C for 30 min. On the other hand, the optimal extraction of the methanolic content from the fruits and stems of *Lysiloma divaricata* was 91.61 ± 0.70 mg GAE/g DM and 212.57 ± 3.62 mg GAE/g DM at 60 °C and 50 °C for 30 min and 90 min, respectively. The optimal temperature for the extraction of this methanolic extract was 40 °C for different extraction times. For all the samples, optimal extraction was achieved for the leaves of *Lysiloma divaricata*. This result agrees with that reported in the literature [37], in which the highest content of polyphenolic compounds is reached in leaves. In addition, time and temperature improved the extraction, resulting in better results for the methanolic system, followed by the ethanolic system, and finally the aqueous system. This was due to the polarity of the solvents.

Furthermore, temperature is an important parameter for determining the stability of phytochemical compounds. The stability of phytochemical compounds has been reported between 25 and 40 °C [38]. However, when these compounds are subjected to an extraction process, high temperatures can enhance the extraction of thermally stable molecules, which increases their solubility in the solvent. However, high temperatures can cause the decomposition of thermostable phenolic compounds [39]. Therefore, extraction temperatures of 40, 50, and 60 °C are maintained because temperatures above 80 °C can adversely affect extraction, causing degradation.

### 3.3. Determination and Quantification of Bioactive Compounds via UPLC MS/MS

**Figure 3:**
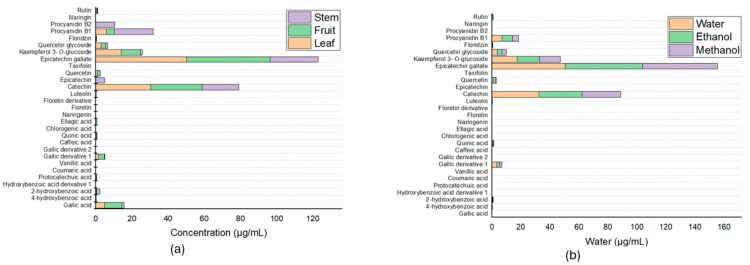
Determination and quantification of bioactive compounds (**a**) in the leaves, fruits, and stems of Lysiloma divaricata and (**b**) using three solvents: water, ethanol/water (30:70 % v/v), and methanol/water (30:70 % v/v) in the leaves of *Lysiloma divaricata*.

The phenolic compounds present in the extracts were tentatively characterized and quantified Via UPLC-MS/MS. When the different fractions were compared in terms of extraction solvent (water/ethanol 70:30 % v/v), phenolic acids and flavonoids were measured, and 27 compounds were determined. Figure 3a shows that the greatest proportion of the compounds were epicatechin gallate, with a retention time of 5.87, with values of 46.19 ± 3.05 μg/mL in the fruit, 50.15 ± 2.82 μg/mL in the leaf, and 26.50 ± 0. 89 μg/mL in the stem.

In addition, gallic acid is one of the six main compounds present in all extracts, besides which has health benefits [12]. These six compounds besides gallic acid are vanillic acid, caffeic acid, quinic acid, chlorogenic acid, and procyanidin B2; these compounds are present in all three fractions, with differences in vanillic acid, which is present only in the branches; caffeic acid, which is not found in the leaves; quinic acid, which is not found in the fruits; chlorogenic acid, which is found only in the stems; ellagic acid, which is found only in the fruits; and procyanidin B2, which is found only in the stems. From these results and those obtained by means of a spectrophotometer and according to the literature, which reports that a large number and variety of phytochemical compounds are present in the leaves, leaf samples were taken to compare the influence of the different solvents used in this work, which revealed that the compounds previously identified in the comparison of the different fractions were preserved; however, the proportions present in each of the extracts changed according to the solvent used, and the results are shown in Figure 3b and Table 2.

### 3.4. Quantification of Gallic Acid by HPLC

HPLC analysis identified gallic acid. Because gallic acid is a natural and stable phenol, it is often used as a reference standard for calibration curves. Phenol measurements are normally expressed as “gallic acid equivalents” (GAE), which means that the total concentration of phenols in the sample is compared to the concentration of a gallic acid solution of known concentration. According to the spectrophotometric method, 12 samples were selected, 6 corresponding to the fractions (leaf, fruit, and stem) of which 3 were selected to have the minimum value and 3 the maximum value of gallic acid; the other 6 included 3 corresponding to the maximum values and 3 corresponding to the minimum values of gallic acid in the samples corresponding to the solvents (water, ethanol/water 30:70 % v/v, methanol/water 30:70 % v/v); some of these samples were duplicated for this reason, and only these samples were selected.

Hence, the importance of quantifying gallic acid in the samples and measuring the concentration of this phenol in the samples, according to the spectrophotometric method, yielded increasingly lower amounts when different solvents were used (water, ethanol/water 30:70 % v/v, and methanol/water 30:70 % v/v) and when different fractions (leaf, fruit, and stem) were used.

**Figure 4:**
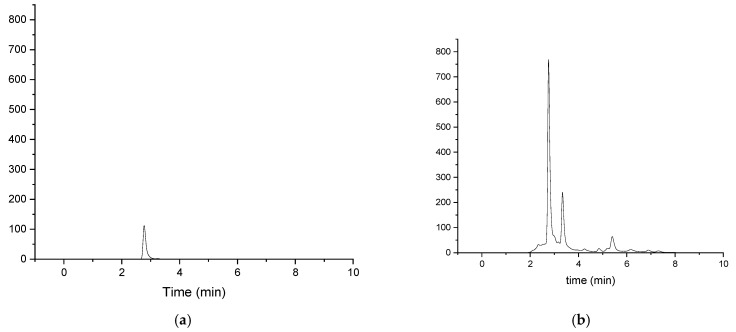
Chromatograms obtained via HPLC for the quantification of gallic acid in *Lysiloma divaricata* samples. (**a**) Standard and (**b**) water-containing leaves at 40 °C for 90 min.

The chromatograms of gallic acid quantified in the *Lysiloma divaricata* extract prepared under different technical conditions are shown in Figure 4. The retention time of gallic acid was 2.7 min. The present results revealed that the methanol/water 30:70 % v/v stem, 40 °C, 90 min solvents and methanol/water 30:70 % v/v stem, 50 °C, 60 min fractions had the lowest gallic acid content, as it was not detected, whereas the highest was that of the water leaf, 40 °C, 90 min fractions sample, with a value of 0.79 ± 0.08 μg of gallic acid/mL, as shown in Table 3. These results may be due to the temperature, time, and solvents used during the extraction process. The samples with the highest and lowest EAGs were taken by spectrophotometry, covering parameters of different extracts according to the solvent and fraction used, showing the importance of identifying the gallic acid content in each of the fractions since, as is known, each of these fractions has a different phytochemical content [37]. As shown in the results, the highest gallic acid content was found in the leaf samples. It is also important to consider the different solvents used for extraction, since, according to [40], their polarity favors the extraction of different phytochemical compounds.

### 3.5. Determination of Antioxidant Activity with DPPH

**Figure 5:**
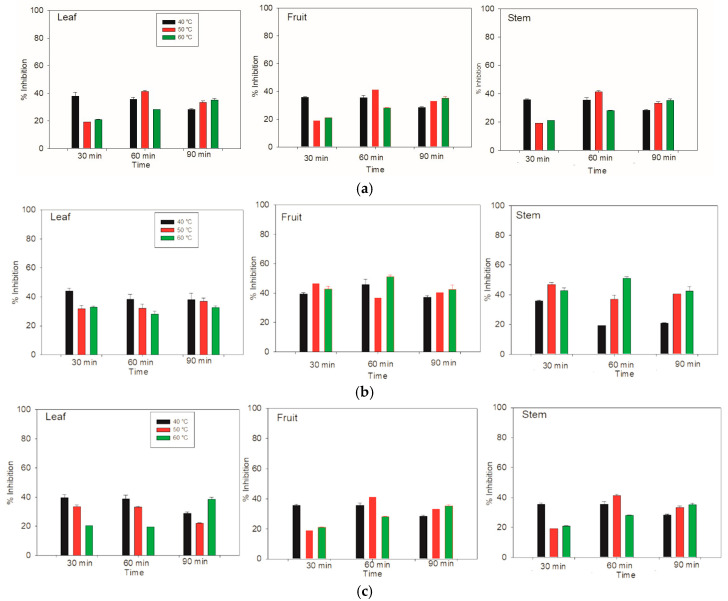
Antioxidant activity was determined via the DPPH method in the leaves, fruits and stems of *Lysiloma divaricata* at three temperatures (40 °C, 50 °C, and 60 °C), three times (30 min, 60 min, 90 min), and three solvents: (**a**) water, (**b**) ethanol/water (30:70 % v/v), and (**c**) methanol/water (30:70 % v/v).

For the aqueous extraction by the DPPH method, the greatest inhibition of the whole system was achieved in the stem and fruit components at 50 °C and 40 °C for 90 min and 60 min, respectively, resulting in an inhibition of over 43% (Figure 5). On the other hand, for the leaf component, the highest inhibition of 41% was achieved at 40 °C for 30 min. Therefore, the stem and fruit compounds have a relatively high affinity for inhibiting the DPPH radical.

In the case of the ethanolic extracts of the leaves at 40 °C, the percentage inhibition was very similar. However, for the fruit, the ethanolic extraction time of 60 min was superior to that of the other times. For the stem, the greatest inhibition at 40 °C occurred at 30 min. On the other hand, the leaf at 50 °C had a similar percentage of inhibition three times. However, in the fruit, the greatest inhibition was reached at 30 min. The inhibition was the same for the 60 and 90 min durations. This behavior was very similar to that of the stem component, with the greatest inhibition of the radical for the fruit and stem occurring at 60 °C for 60 min.

The results of methanolic extraction at 40 °C from the leaves, fruits, and stems of *Lysiloma divaricata* were identical. At 30 and 60 min, the percentage of inhibition was close to 40%, and there was a decrease at 90 min, with values less than 40%. For the leaves at 50 °C and 30 and 60 min, the percentage of inhibition was similar; however, this percentage of inhibition decreased by 90 min. The fruits and stems showed similar behavior, in which at 30 min, there was a lower inhibition of close to 20%. At 60 min, there was an increase in radical inhibition, with values above 40%, and at 90 min, there was a decrease in inhibition. Similar behavior was observed for the leaves heated at 60 °C for 30 and 60 min. At 90 min, greater inhibition of the radical was observed, showing a similar behavior for the three temperatures. In addition, the fruit and stem showed the same ascending behavior as at the other temperatures, according to the time elapsed. These results revealed the greatest inhibition of the fruit at 50 °C for 60 min, followed by that at 40 °C for both 30 min and 60 min.

The greatest inhibition of the DPPH radical activity of the entire system was observed for the stem and fruit, with inhibition percentages of 86.99 ± 1.31 and 86.37 ± 0.30 at 50 °C for 90 min and 40 °C for 60 min, respectively. The third best inhibition was for the stem at 40 °C for 60 min, with an inhibition of 85.06 ± 0.42. For these measurements, we used water as the solvent. According to Taufiq & Sulfiani [41], the polarity of the solvent can affect the antioxidant activity. Thus, the aqueous system reported by Makkiyah et al. [42] presented the highest antioxidant activity, which agreed with the results of Flórez et al. [43]. This research assessed methanol, ethanol and water using nettle leaves (*Urtica dioica*). For example, a polar solvent can have greater antioxidant activity [44], and methanolic extracts can be more effective in eliminating DPPH radicals. On the basis of the research reported in the literature [42], in which four types of solvents (water, acetone, methanol, and ethanol) with different levels of polarity were used, the water/ethanol mixture had the highest antioxidant activity.

### 3.6. Determination of Antioxidant Activity via FRAP

**Figure 6:**
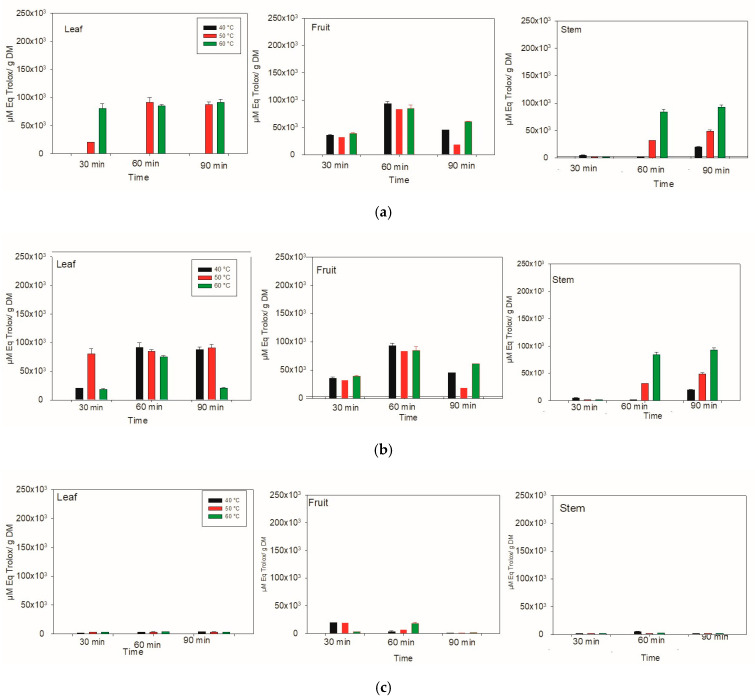
The antioxidant activity was determined via the FRAP method in the leaves, fruits and stems of *Lysiloma divaricata* at three different temperatures (40 °C, 50 °C, and 60 °C), three times (30 min, 60 min, 90 min), and three solvents: (**a**) water, (**b**) ethanol/water (30:70 % v/v) and (**c**) methanol/water (30:70 % v/v).

In the FRAP method, at 40 °C, the greatest capacity to reduce iron ions in the aqueous system was detected in the leaves of *Lysiloma divaricata* at 60 and 90 min, as well as in the fruits at 60 min (Figure 6). Even at a temperature of 50 °C, a high capacity to reduce iron ions was detected in the leaves, as was the case for the fruit at 60 °C. The highest antioxidant activity was reported in the leaf at 60 min, in the fruit at 60 min and in the stem at 60 and 90 min. The highest antioxidant activity was observed in the fruit at 40 °C for 60 min, with a value of 93,112.70 ± 4403.80 μM eq Trolox/g DM, followed by the stem at 60 °C for 90 min, with a value of 92,226.30 ± 4178.80 μM eq Trolox/g DM. Finally, the leaves heated at 40 °C for 60 min presented an antioxidant activity of 91,158 ± 8904.00 μM eq Trolox/g DM, and those heated at 50 °C for 90 min presented an antioxidant activity of 91,061.40 ± 5810.10 μM eq Trolox/g DM.

For the ethanolic system at 40 °C, the highest antioxidant activity was detected in the leaves of *Lysiloma divaricata* after 30 min and 90 min and in the stems of *Lysiloma divaricata* after 30 and 60 min, which decreased for 60 min and then increased for 30 and 90 min. At 50 °C, the highest antioxidant activity (19,586 ± 44.20 μM eq Trolox/g DM) was detected in the leaves after 90 min, followed by the same leaves after 40 °C for 30 min, with a value of 19,1861 ± 14.70. This behavior of antioxidant activity was also reported for stems at different temperatures and times since the values are very close to each other, with values above 15,000 µM Eq Trolox/g DM.

In the methanolic system, the highest antioxidant activity was measured for the fruit of *Lysiloma divaricata* at three temperatures (40 °C, 50 °C, and 60 °C), for which the extraction time was varied. For 40 and 50 °C, 30 min was the best time to achieve better antioxidant activities, with values of 19,745.40 ± 123.90 μM eq Trolox/g DM and 19,726.60 ± 769.70 μM eq Trolox/g DM, respectively. At 60 °C, 60 min was the best time to reach a better antioxidant activity of 18,168.80 ± 2282.70 µM Eq Trolox/g DM.

With the FRAP method, the highest antioxidant activity when water was used as the solvent was obtained for the fruit of *Lysiloma divaricata* at 40 °C for 60 min. With respect to ethanol, the highest antioxidant activity was obtained for the leaf at 50 °C for 90 min. On the other hand, the highest antioxidant activity when methanol was used for the fruit of *Lysiloma divaricata* at 40 °C for 30 min. This result agrees with that obtained by Abu and TV [45], who reported that the aqueous extract had higher values of antioxidant activity than did the ethanolic extracts and concluded that most of the antioxidant compounds were hydrophilic. In addition, these results agree with those reported by Antony and Farid [46], in which the antioxidant activity at 20 °C was 1.7 times greater than the activity observed at 120 °C. These authors suggested that the oxidation of polyphenols at high temperatures can also lead to a loss in antioxidant activity; thus, relatively high temperatures cause a decrease in antioxidant activity.

### 3.7. Determination of Antioxidant Activity with ABTS

**Figure 7:**
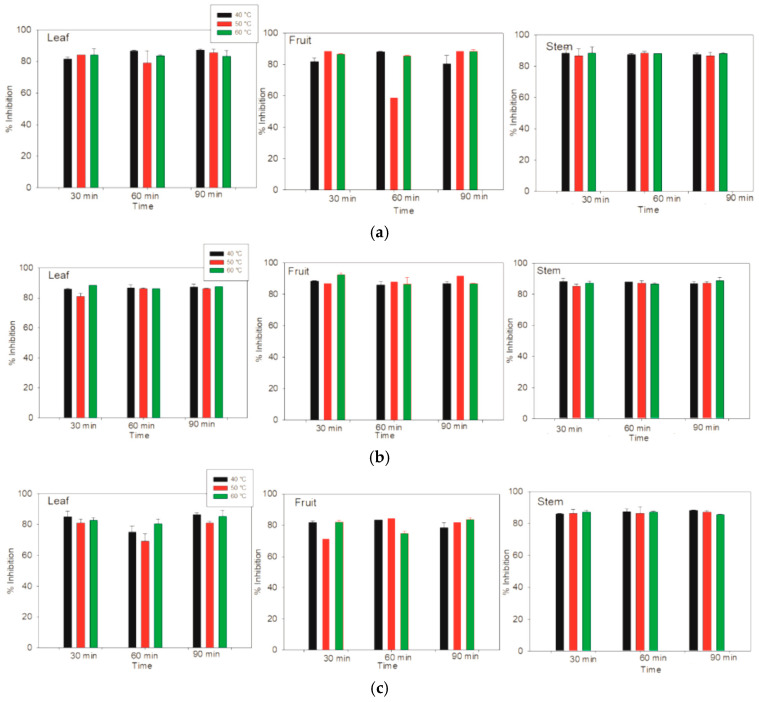
The antioxidant activity of the leaves, fruits and stems of *Lysiloma divaricata* was determined via the ABTS method at three temperatures (40 °C, 50 °C and 60 °C), three times (30 min, 60 min, 90 min), and three solvents: (**a**) water, (**b**) ethanol/water 30:70 % v/v, and (**c**) methanol/water 30:70 % v/v.

On the basis of the antioxidant activity determined via the DPPH method, a high percentage (51.06 ± 1.10) of the fruits of *Lysiloma divaricata* were obtained via ethanol/water (30:70 % v/v) at 60 °C for 60 min. In terms of the antioxidant activity determined by the ABTS method, there were no differences among the samples measured; however, greater similarity was shown for the stem samples. In terms of the three solvents, the three temperatures and the three times differed from those of the other samples, especially the leaf samples; however, these differences did not significantly differ. All the samples achieved values of antioxidant activity between 83% and 87% via the ABTS method, as shown in Figure 7. For FRAP, which is a method based on the measurement of the antioxidant activity of lipophilic and hydrophilic compounds, the highest antioxidant activity, (19,5861 ± 44.19 µM Eq. Trolox/g MS), was obtained for the leaf sample of *Lysiloma divaricata* via ethanol at 50 °C for 90 min.

For the measurement of color by the CIELab system, the parameter *L* represents the luminosity, whereas the parameters a* and b* represent the red, green and yellow-blue color components, respectively [47]. According to Végh et al. [48], color can be an indicator of the antioxidant activity of a product because pigments are associated with antioxidant activity. With respect to the results obtained for the leaf samples, the samples subjected to methanol at 40 °C for 60 min presented greater luminosity, with a value of 20.78, followed by those subjected to methanol at 50 °C for 60 min, with a value of 20.75. Finally, the extraction using ethanol at 60 °C for 30 min achieved a luminosity value of 20.41, whereas the samples with the lowest luminosity (11.85) were subjected to methanolic extraction for 30 min at 60 °C, followed by ethanolic extraction at 90 min, with the temperature varying from 40 °C to 50 °C and luminosity values between 7.20 and 6.50.

According to Su et al. [49], processing and storage cause the oxidation of phenolic compounds due to the activity of polyphenol oxidase, which can lead to darkening and increased residual antioxidant activity. It can affect luminosity and antioxidant activity. On the basis of the research reported by Demiray et al. [50] and Salehi et al. [51], there is a relationship between the parameter *L* and the antioxidant activity of some foods, and higher values of L* are associated with a decrease in antioxidant activity. The red, green component had the highest value (4.78) when water was used as the solvent at 50 °C for 30 min, followed by 60 °C for 60 min. Finally, at 40 °C for 30 min, the value of *a* was 4.48. Considering the research presented by Armijo et al. [52], these values were positively correlated with relatively high intensities, resulting in relatively high antioxidant activity. On the other hand, the lowest value (2.45) of *a* was obtained when ethanol was used at 60 °C for 30 min, followed by the methanol samples at 90 min, both at 60 and 50 °C. The results of the extraction of the fruit and stem samples of *Lysiloma divaricata* are presented in Table 4, Table 5 and Table 6.

## 4. Conclusions

The results of the extraction of phytochemical compounds from different parts (leaf, fruit, and stem) of *Lysiloma divaricata* are reported. The impacts of variations in the temperature, time, solvent, and fraction of the plant were assessed. Using the UPLC-MS/MS method, we identified 27 phytochemical compounds. Using the spectrophotometric method and HPLC, we found that leaf samples presented the highest amount of preserved gallic acid when water was used as the solvent. For the DPPH method, the greatest inhibition was obtained with the fruit that was treated with ethanol/water (30:70 % v/v) at a temperature of 60 °C for 60 min. For the FRAP method, the greatest inhibition was observed with the leaf extract, in which ethanol/water (30:70 % v/v) was used at 50 °C for 90 min. With respect to the ABTS antioxidant activity, there were no significant differences between the samples, which was related to color, indicating that the extraction conditions had an effect.

The extraction of certain compounds, such as gallic acid, epicatechin gallate, and catechin, depended on the temperature, time, solvent, and fraction used, as well as the purpose for which the sample was employed. Thus, the highest gallic acid content was found in the leaf sample, which also provides antioxidant activity. For future applications, it could be considered as a good source of natural antimicrobials due to its biological activity or as a reducing agent for specific compounds, such as AgNO_3_.

## Figures and Tables

**Table 1:** Bromatological analysis of leaf, fruit, and stem samples from *Lysiloma divaricata*.

Samples	Moisture (%)	Ash (%)	Protein (%)	Lipids (%)	Fiber (%)	Carbohydrates (%)
Leaf	5.83 ± 1.26 ^a^	10.80 ± 1.30 ^a^	8.40 ± 0.51 ^a^	4.00 ± 0.25 ^a^	51.75 ± 1.57 ^b^	18.62 ± 2.12 ^b^
Fruit	7.35 ± 0.10 ^a^	4.17 ± 0.04 ^b^	7.26 ±1.00 ^a^	2.01 ± 0.07 ^b^	74.7 ± 3.10 ^a^	3.91 ± 3.31 ^c^
Stem	6.48 ± 1.65 ^a^	5.48 ± 0.04 ^b^	3.47 ± 0.85 ^b^	3.72 ± 0.07 ^a^	53.43 ± 1.97 ^b^	27.10 ± 1.79 ^a^

Different letters (^a^, ^b^, ^c^) within each column indicate significant differences for each variable (*p* = 0.05).

**Table 2:** Phenolic profile of *Lysiloma divaricata* extracts via UPLC-MS/MS.

No.	Compound	Acronym	Retention Time (min)	CAS Number	Main Transitions	λ Max
1	Gallic acid	GaA	1.16	149-91-7	169.15 > 125.05	270.86
2	4-Hydroxybenzoic acid	4-HA	3.22	99-96-7	137.04 > 93.05	253.86
3	2-Hydroxbenzoic acid	2-HA	6.26	69-72-7	153.15 > 83.04	290.86
4	Protocatecholic acid	PA	0.61	99-50-3	153.15 > 109.05	258.86
5	Coumaric acid	CouA	2.19	501-98-4	163.24 > 119.08	308.86
6	Vanillic acid	VA	4.91	121-34-6	167.18 > 152.02	259.96
7	Caffeic acid	CaA	3.70	331-39-5	179.19 > 135.08	321.86
8	Quinic acid	QA	0.57	77-95-2	191.20 > 85.06	254.86
9	Chlorogenic acid	ChA	2.14	327-97-9	353.10 > 191.20	324.86
10	Ellagic acid	EA-Xyl)	3.85	476-66-4	367 > 373	367
11	Hydroxybenzoic derivative 1	2-HA-2	0.64	99-96-7	200 > 300	254
12	Gallic derivative 1	GA-Hex (I)	3.45	149-91-7	230 > 300	256
13	Gallic derivative 2	GA-Hex (II)	5.72	149-91-7	230 > 300	256
14	Naringenin	N	8.97	67604-48-2	270.97 > 150.92	268.86
15	Luteolin	Lut	8.15	491-70-3	250 > 270	270
16	Catechin	Cat	3.48	7295-85-4	289.04 > 245	282.86
17	Epicatechin	epi-C	4.35	490-46-0	288.97 > 245.06	278.86
18	Quercetin	Q	8.17	117-39-5	300.95 > 150.96	366.86
19	Taxifolin	Tax	5.80	480-18-2	470.96 > 272.98	358.86
20	Epicatechin gallate	ECat	5.87	1257-08-5	441.05 > 168.92	265.86
21	Kaempferol 3-O-glucoside	Q-glur	6.59	480-10-4	266 > 366	366
22	Quercetin glycoside	Q-glu	5.96	482-35-9	476.92 > 300.99	355.86
23	Floridzin	Flor	7.28	60-81-1	470.96 > 272.98	271.86
24	Procyanidin B1	PB1	3.21	20315-25-7	470.96 > 272.98	275.86
25	Naringin	Nar	6.8	10236-47-2	579 > 150.96	282.86
26	Rutin	R	5.76	153-18-4	609.04 > 270.94	365.86
27	Floretin	PTN	9.14	60-82-2	272.91 > 166.97	268.86
28	Floretin derivative	FlorD	9.16	60-82-2	272.91 > 166.97	268.86
29	Procyanidin B2	PB2	3.95	29106-49-8	470.96 > 272.98	275.86

**Table 3:** Gallic acid content in leaf, fruit, and stem samples treated with different solvents, showing those with lower and higher contents in each of the systems according to the gallic acid equivalent obtained Via the spectrophotometric method.

Sample	Retention Time (min)	Concentration μg Gallic Acid/mL	Total Phenoles mg Gallic Acid/g DM
Water–Leaf 40 °C 30 min solvents	2.81	0.67 ± 0.02 ^ab^	195.31 ± 3.50 ^c^
Water–Leaf 40 °C 90 min fractions	2.77	0.79 ± 0.08 ^a^	9.26 ± 0.00 ^f^
Water–Fruit 40 °C 60 min solvents	2.76	0.22 ± 0.01^cd^	14.52 ± 2.83 ^e^
Ethanol/water (30:70 % v/v) Leaf-40 °C 60 min solvents	2.76	0.49 ± 0.25 ^abc^	107.39 ± 1.09 ^d^
Ethanol/water (30:70 % v/v) Stem-50 °C 60 min solvents	2.70	0.01 ± 0.00 ^d^	8.38 ± 2.24 ^g^
Methanol/water (30:70 % v/v) Leaf-40 °C 30 min solvents	2.71	0.40 ± 0.05 ^bc^	248.95 ± 7.63 ^a^
Methanol/water (30:70 % v/v) Fruit-40 °C 30 min fractions	2.83	0.20 ± 0.01 ^cd^	213.92 ± 3.09 ^b^

Different letters (^a^, ^b^, ^c^, ^d^, ^e^, ^f^, ^g^) within each column indicate significant differences for each variable (*p* = 0.05).

**Table 4:** Color determination of the leaf sample of *Lysiloma divaricata*.

Time (min)	Temp. (°C)	Solvent	*L*	*a*	*b*	*C*
30	40	Water	15.2 ± 0.1 ^abc^	4.5 ± 0.0 ^ab^	3.9 ± 2.8 ^bcdefgh^	6.1 ± 1.8 ^bcdef^
		Ethanol/water 30:70 % v/v	13.7 ± 0.3 ^abcde^	3.2 ± 0.6 ^defgh^	1.7 ± 2.8 ^fghi^	3.9 ± 1.7 ^efg^
		Methanol/water 30:70 % v/v	18.2 ± 0.4 ^abc^	2.7 ± 0.1 ^fghi^	0.5 ± 0.1 ^hi^	2.7 ± 0.0 ^g^
	50	Water	14.9 ± 0.5 ^abc^	4.8 ± 0.2 ^a^	6.6 ± 1.1 ^abcd^	8.2 ± 1.0 ^abc^
		Ethanol/water 30:70 % v/v	12.2 ± 0.6 ^bcde^	2.9 ± 0.0 ^efghi^	2.1± 0.7 ^fghi^	2.7 ± 0.1 ^efg^
		Methanol/water 30:70 % v/v	17.5 ± 4.1 ^abc^	2.5 ± 0.2 ^hi^	0.8 ± 0.2 ^hi^	3.9 ± 1.4 ^g^
	60	Water	18.5 ± 0.9 ^abc^	4.5 ± 0.1 ^ab^	7.6 ± 3.7 ^ab^	8.8 ± 3.2 ^ab^
		Ethanol/water 30:70 % v/v	11.9 ± 7.9 ^cde^	3.4 ± 0.4 ^def^	3.7 ± 2.0 ^bcdefghi^	2.7 ± 0.1 ^cdefg^
		Methanol/water 30:70 % v/v	20.4 ± 3.0 ^a^	2.6 ± 0.2 ^fghi^	0.4 ± 0.0 ^hi^	5.3 ± 2.9 ^g^
60	40	Water	19.2 ± 0.2 ^abc^	3.4 ± 0.1 ^def^	3.0 ± 0.1 ^defghi^	4.6 ± 0.1 ^defg^
		Ethanol/water 30:70 % v/v	15.2 ± 5.8 ^abc^	2.7 ± 0.0 ^fghi^	1.5 ± 1.1 ^ghi^	3.4 ± 0.9 ^fg^
		Methanol/water 30:70 % v/v	20.8 ± 0.3 ^a^	2.7 ± 0.2 ^fghi^	0.6 ± 0.5 ^hi^	2.8 ± 0.2 ^g^
	50	Water	17.1 ± 0.9 ^abc^	3.8 ± 0.0 ^bcd^	5.3 ± 0.9 ^abcdefg^	6.6 ± 0.9 ^bcde^
		Ethanol/water 30:70 % v/v	19.9 ± 1.1 ^ab^	3.4 ± 0.4 ^def^	0.3 ± 0.3 ^hi^	3.4 ± 0.5 ^fg^
		Methanol/water 30:70 % v/v	20.8 ± 0.2 ^a^	2.5 ± 0.0 ^ghi^	-0.2 ± 0.9 ^i^	2.6 ± 0.1 ^g^
	60	Water	16.3 ± 0.2 ^abc^	4.6 ± 0.2 ^ab^	7.2 ± 0.3 ^abc^	8.5 ± 0.3 ^abc^
		Ethanol/water 30:70 % v/v	19.1 ± 1.1 ^abc^	3.6 ± 0.2 ^cde^	2.2 ± 0.6 ^efghi^	4.3 ± 0.5 ^defg^
		Methanol/water 30:70 % v/v	16.9 ± 2.6 ^abc^	2.5 ± 0.1 ^ghi^	2.4 ± 1.0 ^efghi^	3.5 ± 0.6 ^efg^
90	40	Water	14.5 ± 2.0 ^abcd^	4.5 ± 1.0 ^ab^	5.8 ± 0.9 ^abcdef^	7.4 ± 0.1 ^abcd^
		Ethanol/water 30:70 % v/v	7.2 ± 5.8 ^de^	3.2 ± 0.4 ^defghi^	3.5 ± 1.4 ^bcdefghi^	4.7 ± 1.4 ^defg^
		Methanol/water 30:70 % v/v	19.4 ± 2.1 ^abc^	2.6 ± 0.0 ^fghi^	0.9 ± 0.2 ^hi^	2.8 ± 0.1 ^g^
	50	Water	13.7 ± 5.9 ^abcde^	3.3 ± 0.1 ^defg^	2.6 ± 2.7 ^hi^	4.5 ± 1.5 ^defg^
		Ethanol/water 30:70 % v/v	6.5 ± 0.7 ^e^	3.9 ± 0.7 ^bcd^	6.2 ± 2.3 ^abcde^	7.3 ± 2.3 ^abcd^
		Methanol/water 30:70 % v/v	15.7 ± 1.5 ^abc^	2.4 ± 0.2 ^i^	1.8 ± 0.6 ^fghi^	2.9 ± 0.2 ^fg^
	60	Water	14.3 ± 2.3 ^abcd^	4.5 ± 1.0 ^ab^	9.3 ± 4.9 ^a^	10.4 ± 4.9 ^a^
		Ethanol/water 30:70 % v/v	15.3 ± 2.6 ^abc^	4.3 ± 0.3 ^abc^	3.3 ± 1.7 ^cdefghi^	5.5 ± 0.8 ^cdefg^
		Methanol/water 30:70 % v/v	18.6 ± 3.3 ^abc^	2.4 ± 0.8 ^hi^	0.8 ± 0.9 ^hi^	2.7 ± 0.1 ^g^

Values with the same letter within each variable row are significantly equal according to Tukey’s test (*p* < 0.05).

**Table 5:** Color determination of the fruit sample of *Lysiloma divaricata*.

Time (min)	Temp. (°C)	Solvent	*L*	*a*	*b*	*C*
30	40	Water	19.3 ± 3.0 ^a^	2.6 ± 0.0 ^defg^	−0.4 ± 0.9 ^gh^	2.7 ± 0.1 ^defg^
		Ethanol/water 30:70 % v/v	15.4 ± 5.6 ^abcd^	4.4 ± 0.8 ^a^	5.9 ± 4.2 ^a^	7.6 ± 3.7 ^a^
		Methanol/water 30:70 % v/v	13.0 ± 7.0 ^abcde^	2.6 ± 0.2 ^defg^	1.7 ± 2.3 ^cdefgh^	3.4 ± 1.3 ^cdefg^
	50	Water	7.4 ± 6.2 ^cde^	2.9 ± 0.4 ^bcdefg^	2.9 ± 1.8 ^abcdef^	4.3 ± 0.9 ^cdefg^
		Ethanol/water 30:70 % v/v	18.6 ± 0.1 ^ab^	2.5 ± 0.2 ^efg^	-0.1 ± 0.1 ^fgh^	2.5 ± 0.2 ^efg^
		Methanol/water 30:70 % v/v	16.8 ± 0.5 ^abc^	3.8 ± 0.6 ^abcd^	3.7 ± 1.6 ^abcd^	5.3 ± 1.5 ^abcd^
	60	Water	10.4 ± 3.3 ^bcde^	3.8 ± 1.7 ^abcd^	2.6 ± 2.5 ^cdefgh^	4.7 ± 2.8 ^bcdefg^
		Ethanol/water 30:70 % v/v	15.5 ± 1.5 ^abcd^	2.4 ± 0.1 ^efg^	1.1 ± 0.4 ^defgh^	2.6 ± 0.3 ^efg^
		Methanol/water 30:70 % v/v	14.9 ± 2.6 ^abcde^	2.4 ± 0.3 ^fg^	2.7 ± 0.3 ^cdefgh^	3.6 ± 0.4 ^cdefg^
60	40	Water	16.9 ± 0.2 ^abc^	3.1 ± 0.4 ^bcdefg^	0.1 ± 0.2 ^fgh^	3.1 ± 0.4 ^cdefg^
		Ethanol/water 30:70 % v/v	14.0 ± 5.3 ^abcde^	4.0 ± 0.2 ^ab^	5.8 ± 2.8 ^ab^	7.2 ± 2.4 ^ab^
		Methanol/water 30:70 % v/v	14.4 ± 8.0 ^abcde^	2.4 ± 0.6 ^efg^	1.1 ± 1.1 ^defgh^	2.8 ± 0.0 ^cdefg^
	50	Water	13.6 ± 1.1 ^abcde^	2.5 ± 0.4 ^efg^	1.1 ± 0.1 ^defgh^	2.7 ± 0.4 ^defg^
		Ethanol/water 30:70 % v/v	13.6 ± 9.4 ^abcde^	2.9 ± 0.9 ^bcdefg^	3.4 ± 0.6 ^abcde^	4.6 ± 0.1 ^bcdefg^
		Methanol/water 30:70 % v/v	9.1 ± 2.4 ^cde^	2.8 ± 0.2 ^bcdefg^	3.9 ± 1.3 ^abcd^	4.9 ± 0.9 ^bcde^
	60	Water	13.5 ± 4.7 ^abcde^	3.1 ± 0.9 ^bcdefg^	1.4 ± 1.7 ^cdefgh^	3.5 ± 1.5 ^cdefg^
		Ethanol/water 30:70 % v/v	17.9 ± 0.7 ^ab^	2.8 ± 0.1 ^bcdefg^	1.7 ± 0.0 ^cdefgh^	3.3 ± 0.1 ^cdefg^
		Methanol/water 30:70 % v/v	18.1 ± 1.3 ^ab^	2.1 ± 0.0 ^g^	-0.5 ± 0.2 ^h^	2.2 ± 0.1 ^g^
90	40	Water	8.9 ± 6.7 ^cde^	3.6 ± 1.3 ^abcde^	2.7 ± 1.4 ^bcdefg^	4.7 ± 0.2 ^bcdefg^
		Ethanol/water 30:70 % v/v	17.4 ± 5.7 ^abc^	3.4 ± 0.2 ^abcdef^	3.6 ± 1.8 ^abcd^	5.1 ± 1.4 ^abcde^
		Methanol/water 30:70 % v/v	8.9 ± 2.8 ^cde^	2.4 ± 0.2 ^efg^	2.9 ± 0.9 ^abcdef^	3.8 ± 0.6 ^cdefg^
	50	Water	16.8 ± 3.0 ^abc^	2.8 ± 0.7 ^cdefg^	0.3 ± 0.1 ^efgh^	2.9 ± 0.6 ^cdefg^
		Ethanol/water 30:70 % v/v	18.3 ± 4.4 ^ab^	3.9 ± 0.4 ^abc^	3.6 ± 2.2 ^abcd^	5.4 ± 1.7 ^abc^
		Methanol/water 30:70 % v/v	6.5 ± 2.9 ^e^	2.5 ± 0.4 ^efg^	4.4 ± 0.6 ^abc^	5.0 ± 0.4 ^abcde^
	60	Water	11.6 ± 2.2 ^abcde^	2.3 ± 0.3 ^fg^	1.4 ± 0.3 ^cdefgh^	2.8 ± 0.9 ^cdefg^
		Ethanol/water 30:70 % v/v	14.1 ± 0.8 ^abcde^	3.3 ± 0.2 ^abcdefg^	3.4 ± 1.3 ^abcde^	4.8 ± 1.0 ^bcdef^
		Methanol/water 30:70 % v/v	18.6 ± 0 ^ab^	2.2 ± 0 ^fg^	-0.5 ± 0 ^gh^	2.3 ± 0 ^fg^

Values with the same letter within each variable row are significantly equal according to Tukey’s test (*p* < 0.05).

**Table 6:** Color determination of the stem sample of *Lysiloma divaricata*.

Time (min)	Temp. (°C)	Solvent	*L*	*a*	*b*	*C*
30	40	Water	19.0 ± 0.4 ^a^	11.8 ± 4.9 ^a^	9.1 ± 0.7 ^a^	15.3 ± 7.4 ^a^
		Ethanol/water 30:70 % v/v	18.3 ± 2.4 ^a^	3.1 ± 1.5 ^f^	−0.2 ± 1.4 ^f^	3.3 ± 1.3 ^e^
		Methanol/water 30:70 % v/v	16.1 ± 2.4 ^a^	4.8 ± 0.7 ^ef^	1.1 ± 0.2 ^def^	5.2 ± 1.0 d^e^
	50	Water	17.5 ± 1.9 ^a^	11.0 ± 1.0 ^ab^	8.1 ± 0.8 ^abc^	13.7 ± 1.3 ^a^
		Ethanol/water 30:70 % v/v	12.4 ± 1.5 ^a^	5.7 ± 1.6 ^def^	3.1 ± 4.0 ^cdef^	6.8 ± 3.1 ^bcde^
		Methanol/water 30:70 % v/v	18.3 ± 5.4 ^a^	3.8 ± 0.1 ^f^	0.7 ± 0.4 ^f^	3.9 ± 0.3 ^de^
	60	Water	15.8 ± 1.9 ^a^	11.3 ± 1.9 ^ab^	9.7 ± 4.7 ^a^	14.9 ± 4.5 ^a^
		Ethanol/water 30:70 % v/v	13.7 ± 2.3 ^a^	5.5 ± 1.7 ^ef^	3.8 ± 4.6 ^cdef^	6.8 ± 3.6 ^bcde^
		Methanol/water 30:70 % v/v	13.9 ± 0.7 ^a^	5.2 ± 0.0 ^ef^	4.3 ± 0.1 ^bcdef^	6.7 ± 0.1 ^bcde^
60	40	Water	16.5 ± 1.0 ^a^	10.2 ± 1.6 ^abc^	8.8 ± 3.1 ^ab^	13.6 ± 3.3 ^a^
		Ethanol/water 30:70 % v/v	12.2 ± 2.2 ^a^	4.2 ± 1.4 ^f^	2.6 ± 0.6 ^def^	5.6 ± 3.1 ^de^
		Methanol/water 30:70 % v/v	20.4 ± 1.1 ^a^	4.6 ± 0.2 ^f^	0.3 ± 0.4 ^f^	4.6 ± 0.2 ^de^
	50	Water	16.4 ± 0.8 ^a^	8.6 ± 0.9 ^bcd^	6.2 ± 1.9 ^abcde^	10.6 ± 1.8 ^abc^
		Ethanol/water 30:70 % v/v	17.4 ± 6.4 ^a^	4.1 ± 0.1 ^f^	1.6 ± 2.3 ^def^	4.6 ± 0.9 ^de^
		Methanol/water 30:70 % v/v	12.8 ± 5.3 ^a^	5.0 ± 1.3 ^ef^	3.3 ± 2.9 ^cdef^	6.1 ± 2.7 ^cde^
	60	Water	18.4 ± 0.7 ^a^	7.7 ± 0.4 ^cde^	3.9 ± 0.7 ^bcdef^	8.6 ± 0.7 ^bcd^
		Ethanol/water 30:70 % v/v	19.9 ± 2.3 ^a^	3.4 ± 0.2 ^f^	-0.4 ± 0.0 ^f^	3.5 ± 0.2 ^e^
		Methanol/water 30:70 % v/v	16.7 ± 2.6 ^a^	4.6 ± 0.7 ^f^	1.8 ± 1.0 ^def^	4.9 ± 1.0 ^de^
90	40	Water	19.9 ± 0.2 ^a^	9.2 ± 2.4 a^bc^	6.3 ± 1.5 ^abcde^	11.2 ± 2.8 ^ab^
		Ethanol/water 30:70 % v/v	18.3 ± 2.5 ^a^	5.4 ± 0.4 ^ef^	1.1 ± 0.2 ^f^	5.5 ± 0.3 ^de^
		Methanol/water 30:70 % v/v	11.8 ± 5.5 ^a^	5.6 ± 0.6 ^ef^	3.1 ± 2.1 ^cdef^	6.5 ± 1.5 ^bcde^
	50	Water	17.8 ± 1.2 ^a^	8.9 ± 1.5 ^abc^	6.5 ± 0.6 ^abcd^	11.0 ± 1.6 ^ab^
		Ethanol/water 30:70 % v/v	18.2 ± 3.8 ^a^	4.5 ± 0^4 f^	1.3 ± 0.8 ^ef^	4.8 ± 0.6 ^de^
		Methanol/water 30:70 % v/v	16.1 ± 1.0 ^a^	4.1 ± 0.1 ^f^	1.5 ± 0.5 ^def^	4.4 ± 0.3 ^de^
	60	Water	17.4 ± 1.0 ^a^	5.9 ± 0.2 ^def^	4.5 ± 0.8 ^bcdef^	7.5 ± 0.6 ^bcde^
		Ethanol/water 30:70 % v/v	21.5 ± 0.7 ^a^	4.1 ± 0.0 ^f^	0.3 ± 0.0 ^f^	4.1 ± 0.0 ^de^
		Methanol/water 30:70 % v/v	17.8 ± 6.2 ^a^	4.4 ± 0.5 ^f^	1.7 ± 2.0 ^def^	4.9 ± 1.1 ^de^

Values with the same letter within each variable row are significantly equal according to Tukey’s test (*p* < 0.05).

## Data Availability

Data are contained within the article.
